# A Method for Efficient Loading of Ciprofloxacin Hydrochloride in Cationic Solid Lipid Nanoparticles: Formulation and Microbiological Evaluation

**DOI:** 10.3390/nano8050304

**Published:** 2018-05-06

**Authors:** Rosario Pignatello, Antonio Leonardi, Virginia Fuochi, Giulio Petronio Petronio, Antonio S. Greco, Pio Maria Furneri

**Affiliations:** 1 Section of Pharmaceutical Technology, Department of Drug Sciences, University of Catania, 95125 Catania, Italy; aleonardi@locatetherapeutics.com (A.L.); antonio87251@gmail.com (A.S.G.); 2 NANO-i, Research Centre on Ocular Nanotechnology, University of Catania, 95125 Catania, Italy; 3 Section of Microbiology, Department of Biomedical and Biotechnological Sciences, BIOMETEC, University of Catania, 95125 Catania, Italy; virginia.fuochi@gmail.com (V.F.); gpetroniopetronio@gmail.com (G.P.P.); furneri@unict.it (P.M.F.)

**Keywords:** SLN, triethylamine, positive charge, DDAB, antimicrobial activity, drug encapsulation

## Abstract

The aim of the study was the production of solid lipid nanoparticles (SLN) loaded with ciprofloxacin (CIP) through two different production techniques, quasi-emulsion solvent diffusion (QESD) and solvent injection (SI). In order to efficaciously entrap the commercial salt form (hydrochloride) of the antibiotic in these lipid systems, a conversion of CIP hydrochloride to the free base was realized in situ, through the addition of triethylamine. To ensure physical stability to the carriers over time and ameliorate the interaction with bacterial cell membranes, positively charged SLN were produced by addition of the cationic lipid didecyldimethylammonium bromide (DDAB). Homogeneous SLN populations with a mean particle sizes of 250–350 nm were produced by both methods; drug encapsulation was over 85% for most samples. The SLN were physically stable for up to nine months both at 4 °C and 25 °C, although the former condition appears more suitable to guarantee the maintenance of the initial particle size distribution. As expected, CIP encapsulation efficiency underwent a slight reduction after nine months of storage, although the initial high drug content values would ensure a residual concentration of the antibiotic in the SLN still appropriate to exert an acceptable antibacterial activity. Selected SLN formulations were subjected to an in vitro microbiological assay against different bacterial strains, to verify the effect of nanoencapsulation on the cell growth inhibitory activity of CIP. In general, CIP-SLN produced without DDAB showed MIC values for CIP comparable to those of the free drug. Conversely, addition of increasing percentages of the cationic lipid, reflected by a progressive increase of the positive value of the Zeta potential, showed a variety of MIC values against the various bacterial strains, but with values 2–4 order of dilution lower than free CIP. An hypothesis of the effect of the cationic lipid upon the increased antibacterial activity of CIP in the nanocarriers is also formulated.

## 1. Introduction

Ciprofloxacin (CIP) is a broad spectrum bactericidal antibiotic highly effective against Gram-positive and Gram-negative bacteria, frequently used in urinary tract infections [1], respiratory infections [2], otitis media treatment [3], and in external ocular infections [4,5]. CIP is also used in cases of sexually transmitted bacteria, sepsis, and legionella.

As other molecules of quinolones class, CIP acts by inhibiting DNA gyrase and topoisomerase IV. This action very selective on these two types of enzymes is due to the remarkable conservation of protein sequences between the DNA gyrase subunit A (*gyr*A) and topoisomerase IV subunit C (*par*C) in the quinolone resistance determining region (QRDR), present in bacteria.

In recent years, progressively more cases of resistance to fluoroquinolones have been registered [6,7]. Resistance is mediated mainly by spontaneous mutations in the QRDR of *gyr*A and *par*C genes, causing reduced drug accumulation [8].

Moreover, to reach their targets, quinolones must cross the cell wall and cytoplasmic membrane of Gram-positive bacteria and an additional outer membrane barrier in the case of Gram-negative bacteria, so the risk of resistance can be due even to a decreased uptake or/and an increased efflux [9].

In order to counter the big problem of bacterial resistance to drugs, as well as improve the pharmacokinetic and pharmacodynamics properties of antibacterial drugs, nanotechnology has developed many supra-molecular structures, in which the active compound is embedded in polymer- or lipid-based nanostructures. Among the more promising nanosized drug carriers, solid lipid nanoparticles (SLNs) are being studied in the last years in many therapeutic fields, including the delivery of antibacterial, antifungal, and antiviral drugs [10,11,12,13,14,15].

SLNs are colloidal carrier systems composed of one or more lipids, solid at room conditions, coated by hydrophilic surfactant(s) [16,17]. The term ‘lipid’ is used in a broader sense and includes triglycerides, cetyl palmitate, alkanoic acids, and various synthetic or naturally occurring lipophilic materials, all however characterized by a high level of biocompatibility [18,19]. The main advantages of SLN versus other colloidal drug carriers include the possibility of achieving a controlled drug release, while increasing the physicochemical stability of loaded drugs, both lipophilic and hydrophilic compounds [20]. Additionally, problems related to industrial production scale-up, sterilization, and mid-term storage are reduced with respect to liposomes and polymeric nanocarriers [21,22,23,24].

Some CIP-loaded polymeric and lipid nanoparticles have been described in recent years. Most works dealt with the physicochemical characterization of the nanocarriers [25,26,27,28,29], and only few papers report an in vitro microbiological evaluation of nanoencapsulated CIP [30,31]. Furthermore, in most cases the drug was used as the hydrochloride salt (CIP HCl), the commercial soluble form used in conventional clinical dosage forms, but that is not specifically suitable for inclusion in lipid-based carriers. Sharma et al. reported the employment of CIP free base to obtain lipid nanoparticles with an encapsulation efficiency close to 85% when the antibiotic was loaded alone, or between 55–65% if CIP was loaded in combination with another drug [32].

Therefore, one of the aims of the present study was to optimize the loading of CIP HCl, as model of a water soluble drug salt, through an in situ production of the free base induced by addition of stoichiometric amount of the liposoluble organic base trimethylamine (TEA).

Furthermore, since the method used for the preparation of SLN is a critical step in obtaining a suitable nanoparticle size and homogeneity, along with other technological features such as stability, drug loading, and release, we assessed two different techniques, chosen among those commonly proposed for SLN production [17]: a solvent injection (SI) procedure and the quasi-emulsion solvent diffusion method (QESD). SI presents numerous advantages compared to other methods, including an easy and rapid manufacture, because it does not require sophisticated or dedicated instrumentations and tools [33,34]. QESD, originally proposed for producing polymeric nanoparticles [35,36,37], has been applied in our lab also for lipid-based colloidal systems [38,39,40,41].

In particular, the use of non-toxic organic solvents like acetone or ethanol (ICH class 3: solvents with low toxic potential), combined with low working temperatures were exploited in both methods in the view of a future industrial production of these systems. This first note will present the experimental data refereed to SLN prepared by the SI method.

## 2. Results and Discussion

### 2.1. Preformulation Studies

Preliminary studies were directed to establish the solubility of CIP hydrochloride in different organic solvents. The solubility of the drug is a key factor, because the methodology used for the preparation of nanoparticles requires that the drug is dissolved in an organic solvent, which in turn must be miscible with the aqueous phase. For the sake of a future industrial scaling-up of these methodologies, we chose only low-toxicity (ICH class 3) solvents, such as ethanol or acetone.

From the preliminary tests and according to literature data, CIP hydrochloride resulted poorly soluble in acetone and isopropanol, highly soluble in water and soluble ethanol, with values of 0.12 mg/mL at 25 °C and of about 0.2 mg/mL at 36 °C in the latter solvent [42]. Furthermore, since literature data indicate that CIP base has a better solubility profile in acetone compared to ethanol [43], the method was further optimized by using the former solvent.

To enhance the encapsulation of CIP as the free base (e.g., in a lipophilic form) starting from the commercial hydrochloride salt (hydrophilic form), an in situ acid–base shift method was used, that made possible to solubilize the drug in the organic solvents needed for the production of nanoparticles. The strategy consisted in the addition of calculated amounts of TEA to the acetone suspension of CIP hydrochloride, before the dropwise addition of the latter into the aqueous phase. Also, TEA has been recently included by ICH in the Class 3 solvent list [44].

As formulation variables, the concentration of CIP (100 or 500 µg/mL), DDAB (0 to 0.15% by weight), and TEA were investigated (Table 1 and Table 2). To determine the minimum molar ratio (volume) of TEA required to efficiently convert the hydrochloride salt into CIP base, experiments were performed with increasing aliquots of TEA (5 µL by time). In particular, two drug/TEA molar ratios were considered as the most interesting, i.e., 18:1 and 3.5:1. Nevertheless, to formulate the SLN systems, we decided to use the former molar ratio, since the obtained nanoparticle batches gave better results in terms of Z-ave and size homogeneity.

A batch of empty (blank) SLN was also produced with the composition gathered in Table 3.

### 2.2. Characterization of SLN

The photon correlation spectroscopy (PCS) characterization of the SLNs indicated a mean particle size between 270 and 350 nm. The PdI values ranged between 0.25 and 0.34, indicating that the produced nanoparticle populations in most cases have a good degree of homogeneity (Table 4).

**Figure 1:**
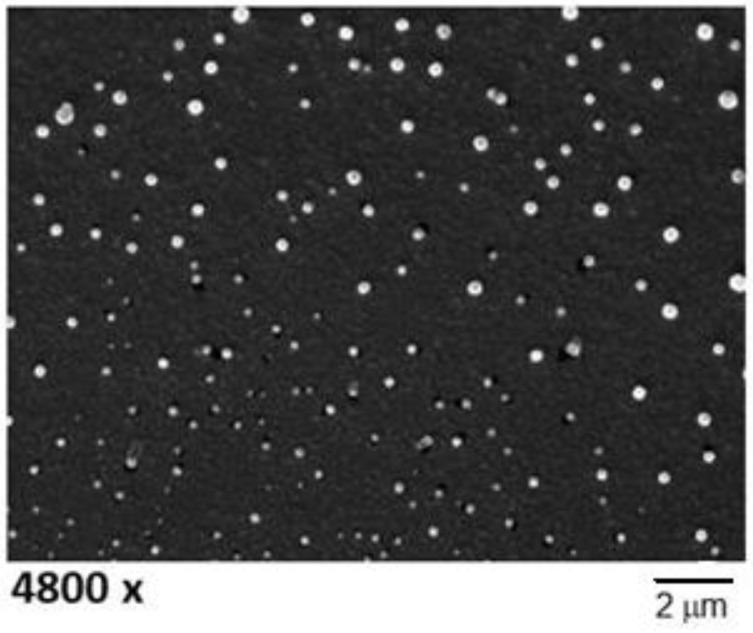

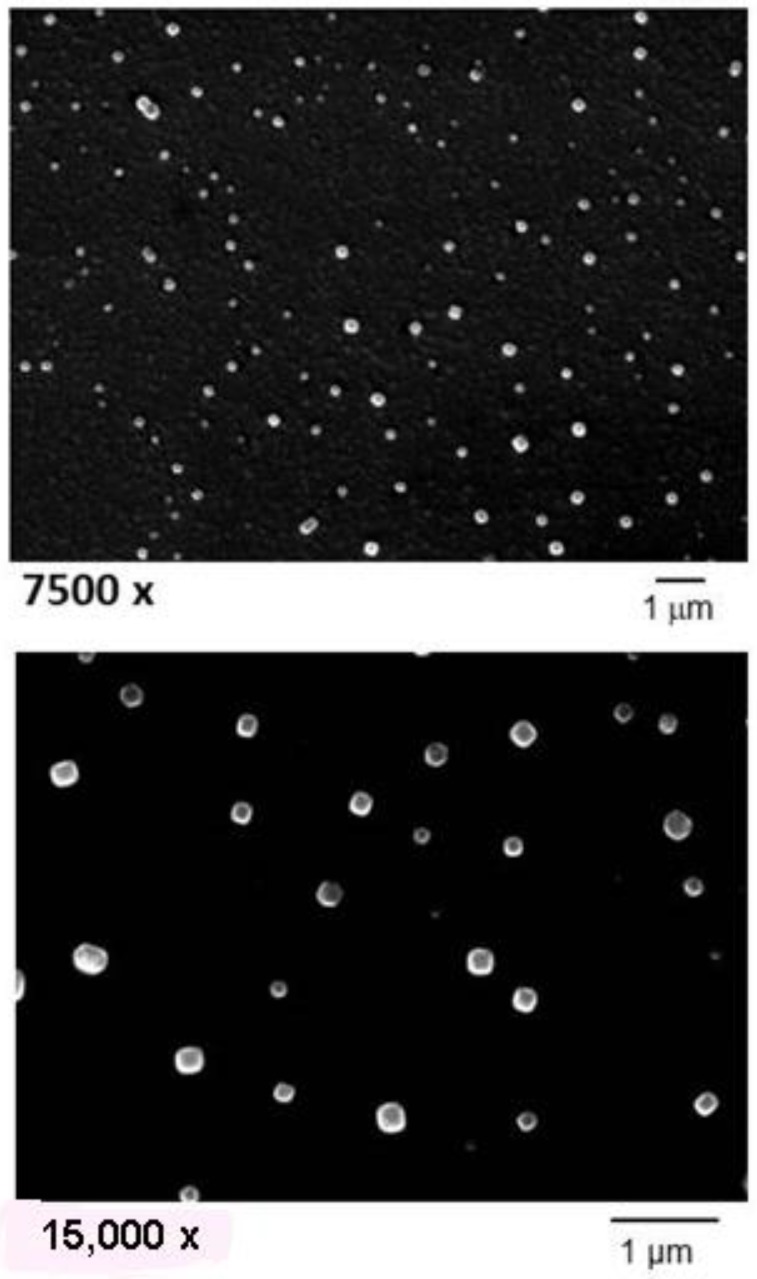
Scanning electron micrographs, at various enlargements, of CIP-loaded cSLN batches C1Si (**top**) and C6Si (**middle** and **bottom**).

Scanning electron microscopy images micrographs of CIP-cSLN show a spherical shape and a mean particle size consistent with the values measured by PCS (Figure 1).

The surface charge was of course affected by the concentration of DDAB: the nanoparticles that did not contain the cationic lipid showed a negative Zeta potential (around −40 mV), which became progressively more positive with increasing concentrations of DDAB. The drug encapsulation efficiency was relatively high for all the SLN formulations, with values between 78 and 99%. Blank (unloaded) cSLN showed a mean size of 280 nm (PdI = 0.292), and a Zeta potential close to +60 mV (Table 5).

### 2.3. Stability Tests

Some SLN batches were stored at two different temperatures (+4 and +25 °C) for up to nine months; periodically, they were re-evaluated for Z-ave, PdI, and zeta potential, and compared to the initially registered values. Experimental data are reported as Supplementary Material to this paper.

Stability studies indicated that all the formulations are quite stable in both conditions, with the exception of formulations C4Si and particularly C3Si, which were stable at 4 °C but at room conditions showed a clear tendency to a progressive size growth or aggregation of the nanoparticles. As a conclusive suggestion, however, storage in a refrigerator or anyhow at a controlled temperature below 25 °C could be advised for these systems.

The zeta potential values remained almost unchanged, with respect to those measured at the production time, along the whole storage period (data not shown). This behavior, similar to that observed for the analogous erythromycin-loaded SLN [15], suggests that the chemical composition of the lipid matrix was maintained in the tested storage conditions, otherwise the loss of S100 and especially DDAB from the nanoparticles would have progressively reduced their surface charge.

Determination of EE% after nine months at both the temperature conditions indicated that CIP loading underwent a slight reduction (Table 4), as expected for this kind of nanocarrier as a consequence of a partial drug expulsion during reorganization of the lipid matrix. The average drug leakage ranged between 10–15% in the samples stored at 4 °C, and around 25% upon storage at r.t. However, the initial high drug content values would ensure a residual concentration of the antibiotic in the SLN still appropriate to exert an acceptable antibacterial activity.

### 2.4. Microbiological Assay

The MIC values of the prepared SLN batches containing 100 µg/mL CIP (Table 1), against different bacterial strains, including Gram-positive and Gram-negative bacteria, are shown in Table 6. The results (not shown) relative to the corresponding batches containing 500 mg/mL of the antibiotic (cf. Table 2) were almost superimposable. Experimental data demonstrated that the nanoparticles produced without DDAB had MIC values comparable to free CIP, as reported by quality control ranges in CLSI [45]. Conversely, the systems containing the cationic lipid (batches C2Si, C3Si, C4Si, as well as C6Si, C7Si and C8Si (not shown)), showed MIC values lower than the free antibiotic against almost all the tested bacterial species. Such a behavior can be ascribed to the lipid nature and positive charge of these nanoparticles that allowed a better interaction with the bacteria surface and penetration through their cell wall. The growth inhibitory activity in fact tended to grow with the increasing positive charge, and was more evident against Gram-negative bacteria, whose cells possess an outer phospholipid membrane covering the cell wall.

Such a feature has been demonstrated recently in a similar study on erythromycin-loaded cSLN [15]. A direct ‘cytotoxic effect’ of DDAB on the bacteria cells cannot be dismissed, since it can be hypothesized that DDAB negatively affect the integrity of the cell membrane; a similar phenomenon has been documented for other quaternary ammonium compounds, although at very high concentrations, anyway much greater than those present in the tested SLN formulations. Therefore, it is correct to postulate that, at the concentrations of DDAB used for the production of these cSLN, a real gain to the antibacterial potency of CIP was given from their positive charge.

Additional microbiological experiments on different blank and loaded SLN formulations—in the presence of increasing concentrations of DDAB—are ongoing, to better try to distinguish the cytotoxic effect of the cationic lipid from the advantageous effects that the positive charge of the resulting SLN could give to the antibacterial activity of loaded antibiotics.

Comparison of our experimental results with literature data on CIP-loaded polymeric and lipid nanocarriers was not easy in terms of effect of nanoencapsulation on the in vitro antibacterial activity of this antibiotic, regardless of the conclusions which the authors reached. This is mainly due to the fact that not all the papers use the same method to express the in vitro bacterial growth inhibitory activity, ranging for instance from presentation of MIC values (as in the present study) to plate diffusion test. The heterogeneity of data in literature and the lack of standardization of in vitro experiments is unfortunately common to many papers in the field of antibacterial drug delivery [13]. However, among the most recent published articles, the encapsulation of CIP, as free base or hydrochloride salt, in polymeric nanoparticles or SLN generally seems to attain an enhancement of the in vitro antibacterial activity, compared to the free drug. This result has been, for instance, ascribed to a more efficacious delivery of the antibiotic inside bacterial cells and to a higher stability of the encapsulated drug in chitosan nanoparticles [45]. SLN loaded with CIP have been studied by plate diffusion testing against *S. aureus* and *P. aeruginosa*. Although, from the published results, it was not possible to evaluate the inhibition zone, the authors however concluded that the nanoparticle formulation showed an higher antibacterial effect than the neat drug [31].

The antimicrobial activity against *Mycobacterium avium* complex in human macrophages was almost doubled on a log scale by loading CIP in polyisobutylcyanoacrylate (PIBCA) nanoparticles, compared with a drug solution. However, the efficiency was much lower than expected, strongly limited by the cytotoxicity of the polymeric material [46]. Conversely, encapsulation of CIP in polyethylbutylcyanoacrylate (PEBCA) nanoparticles did not change its MIC and minimal bactericidal concentration (MBC) values against *S. Typhimurium*, compared with the free drug [47].

In these regards, an interesting consideration concerns the differential results often observed between the in vitro and in vivo assays. It is not uncommon that, due to their slow and prolonged drug release features, nanocarriers in vitro present a lower activity than the free antibiotics, while in vivo they can show a longer duration of the activity (e.g., [48]) and an improved PKs profile, especially when the drug was carried in liposomes [49,50,51].

### 3.1. Material and Methods

DDAB, CIP hydrochloride, Tween^®^ 80, TEA and solvents were purchased from Sigma-Aldrich Chimica srl (Milan, Italy). Softisan^®^ 100 (S100) was kindly donated by IOI Oleo GmbH (Hamburg, Germany). HPLC-grade water was a Merck product (VWR, Milan, Italy).

### 3.2. Preparation of the SLN by Solvent Injection

The procedure involved the preparation of an aqueous phase (10 mL), consisting of water and Tween 80 (0.25%, w/v) and of a separate organic phase, consisting of S100 (100 mg), TEA, DDAB, and CIP hydrochloride in acetone (2.6 mL) (see Table 1 and Table 2 for the weight ratios). Both phases were warmed at 36 °C (the melting temperature of the lipid) under mild stirring, thereafter the organic phase was added dropwise to the aqueous phase by a plastic syringe connected to a G-23 needle. The obtained mixture was then stirred overnight at room temperature at 700 rpm, to ensure a complete evaporation of the organic solvent. The resulting milky suspension was finally bath-sonicated for 20 min (Branson 5002, Branson Ultrasonics, Danbury, USA) at room temperature and at 20 W.

Unloaded (blank) cSLN were produced similarly without addition of CIP (Table 3).

### 3.3. Characterization of SLN

The prepared SLN batches were subjected to PCS analysis using a Nanosizer ZS90 (Malvern Panalytical Ltd, Malvern, UK) connected to a PC running the dedicated PCS v1.27 software. To measure the mean size (Z-ave) and polydispersity index (PdI), an aliquot of each sample was diluted 10-fold with HPLC-grade water and placed in a glass cuvette; measurements were done by a laser beam at a wavelength of 633 nm. The reported values are the mean ± SD of 90 measurements (3 sets of 10 measurements in triplicate). The zeta potential (ZP) was determined by electrophoretic light scattering with the same instrument. Up to 100 measurements on each sample were registered at room temperature, to calculate the electrophoretic mobility and, using the Smoluchowski constant (Ka) with a value of 1.5, the corresponding ZP values.

The spectrophotometric UV analysis was carried out by a Shimadzu UV-1601 instrument (Shimadzu Italia, Milan, Italy). A calibration line (*r*^2^ = 0.9997) for CIP hydrochloride in water was made at 206.0 nm, in a 1–100 µg/mL drug concentration range, obtained by diluting a stock solution (1 mg/10 mL) with appropriate volumes of water for HPLC.

#### 3.3.1. Scanning Electron Microscopy

SLN suspensions were placed on a 200-mesh formvar copper grid and sputter coated with a 5 nm gold layer using an Emscope SM 300. A Hitachi S-4000 field emission scanning electron microscope (Hitachi Ltd., Tokyo, Japan) was used for the observations (acceleration voltage 12 KV, spot 2.5).

#### 3.3.2. Determination of Encapsulation Efficiency (EE%) and Drug Loading (DL)

One mL of each SLN suspension was placed in a Whatman Vectaspin^®^ 20 tube, equipped with a 0.45-μm pore size polypropylene membrane filter (Sigma-Aldrich srl, Milan, Italy). The tubes were ultracentrifuged (IEC CENTRA MP4R) at 4400 rpm and 10 °C for 20 min. Aliquots of the supernatant from the bottom of the device, containing the amount of drug that had not been incorporated in the lipid particles, were withdrawn, diluted 10-fold with HPLC grade water and analyzed by UV analysis at 206 nm. The encapsulation efficiency for each sample was calculated as

EE% = Co − Cw/Co × 100

where Co and Cw are the initial amount of CIP hydrochloride and the amount of drug found in the supernatants respectively. Drug loading was calculated from the above data as the µg of CIP present in one mL of nanoparticle suspension. Each determination for made in three to four replicates.

#### 3.3.3. Stability

An aliquot of each formulation was stored in closed amber glass vials at room temperature (25 ± 2 °C) or at 4 ± 1 °C. Physicochemical and technological parameters were measured every 30 days for nine months.

### 3.4. Antimicrobial Assay

All SLN formulations have been investigated for their antibacterial activity. *Escherichia coli* ATCC 25922, *Pseudomonas aeruginosa* ATCC 27853, *Enterococcus faecalis* ATCC 29212, and *Staphylococcus aureus* ATCC 29213 strains were studied because the MIC range for CIP is available [52]. The values of MIC, defined as the lowest concentration of CIP that inhibited visible bacterial growth at 37 °C after overnight incubation, were measured by microdilution method according to CLSI M100S [52]. Each plate was prepared by including a positive control for growth (C+) and negative control of sterility (C−). Each formulation was tested six times against each bacterial strains; the same experiment was repeated on a different day to ensure reproducibility [53].

## Figures and Tables

**Table 1:** Composition (%, *w*/*v*) of SLN loaded with 100 µg/mL of drug.

Component	C1Si	C2Si	C3Si	C4Si
CIP HCl	0.01	0.01	0.01	0.01
S100	1	1	1	1
TEA	210 µL	210 µL	210 µL	210 µL
DDAB	0	0.05	0.10	0.15
TWEEN^®^ 80	0.25	0.25	0.25	0.25

**Table 2:** Composition (%, *w*/*v*) of SLN loaded with 500 µg/mL of drug.

Component	C5Si	C6Si	C7Si	C8Si
CIP HCl	0.05	0.05	0.05	0.05
S100	1	1	1	1
TEA	1 mL	1 mL	1 mL	1 mL
DDAB	0	0.05	0.10	0.15
TWEEN^®^ 80	0.25	0.25	0.25	0.25

**Table 3:** Composition (%, *w*/*v*) of blank SLN.

Blank Sample	S100	TWEEN^®^ 80	DDAB
C0Si	1	0.25	0.10

**Table 4:** Characterization of the SLNs obtained by the SI method. Reported values are the mean ± S.D. of at least three replicates.

Sample	Size (nm)	PdI	ZP (mV)	EE%	Drug Content (μg/mL)	Drug Content (μg/mL) after 9 Months at 4 °C	Drug Content (μg/mL) after 9 Months at 25 °C
C1Si	353.8 ± 19.24	0.337 ± 0.019	−39.3 ± 1.35	91.1 ± 5.11	91.09 ± 5.11	79.19 ± 6.01	67.11 ± 3.99
C2Si	311.7 ± 4.16	0.233 ± 0.010	+18.7 ± 5.53	88.7 ± 9.98	88.67 ± 9.98	67.65 ± 4.44	66.43 ± 12.00
C3Si	345.0 ± 11.45	0.340 ± 0.052	+35.1 ± 0.81	86.1 ± 1.24	86.11 ± 1.24	69.33 ± 11.11	63.33 ± 9.91
C4Si	315.0 ± 1.51	0.323 ± 0.014	+46.1 ± 0.46	82.9 ± 5.55	82.90 ± 5.55	65.02 ± 4.98	56.43 ± 4.46
C5Si	309.0 ± 6.94	0.257 ± 0.006	−41.9 ± 0.46	93.0 ± 8.01	465.00 ± 37.24	411.66 ± 22.12	357.10 ± 22.00
C6Si	272.0 ± 6.03	0.271 ± 0.081	+32.8 ± 0.70	90.3 ± 3.99	451.50 ± 18.01	411.02 ± 23.91	366.20 ± 23.98
C7Si	285.9 ± 17.91	0.232 ± 0.410	+46.7 ± 0.56	87.7 ± 3.49	438.52 ± 15.30	399.42 ± 33.01	334.22 ± 11.98
C8Si	305.2 ± 5.89	0.268 ± 0.046	+50.5 ± 1.71	89.0 ± 7.12	444.98 ± 31.68	395.22 ± 22.58	345.11 ± 27.98

**Table 5:** Characterization of the blank SLN obtained by SI technique.

Blank Sample	Size (nm)	PdI	ZP (mV)
C0Si	279.1 ± 1.55	0.292 ± 0.030	+58.8 ± 7.51

**Table 6:** MIC values (μg/mL) of cSLN (CIP concentration: 100 μg/mL).

Strain	CIP	C1Si (No DDAB)	C2Si (DDAB: 0.5 mg/mL)	C3Si (DDAB: 1 mg/mL)	C4Si (DDAB: 1.5 mg/mL)
*E. coli* ATCC 25922	≤0.004	≤0.004	0.02	0.01	0.01
*P. aeruginosa* ATCC 27853	1	1	0.6	0.6	0.6
*S. aureus* ATCC 29213	0.5	0.5	0.15	0.03	0.02
*E. faecalis* ATCC 29212	0.5	0.5	0.3	0.06	0.03

## Supplementary Materials

The following data are available online at http://www.mdpi.com/2079-4991/8/5/304/s1, Figures S1 to S8: stability data of CIP-SLN.
