# Supplementary material for: A Method for Efficient Loading of Ciprofloxacin Hydrochloride in Cationic Solid Lipid Nanoparticles: Formulation and Microbiological Evaluation
Source: Nanomaterials (Basel). 2018 May 6;8(5):304. doi: 10.3390/nano8050304 (PMC5977318; doi:10.3390/nano8050304)
Supplement: Supplementary file 1 [file nanomaterials-08-00304-s001.zip › nanomaterials-281888-supplementary.pptx]

## Slide 1
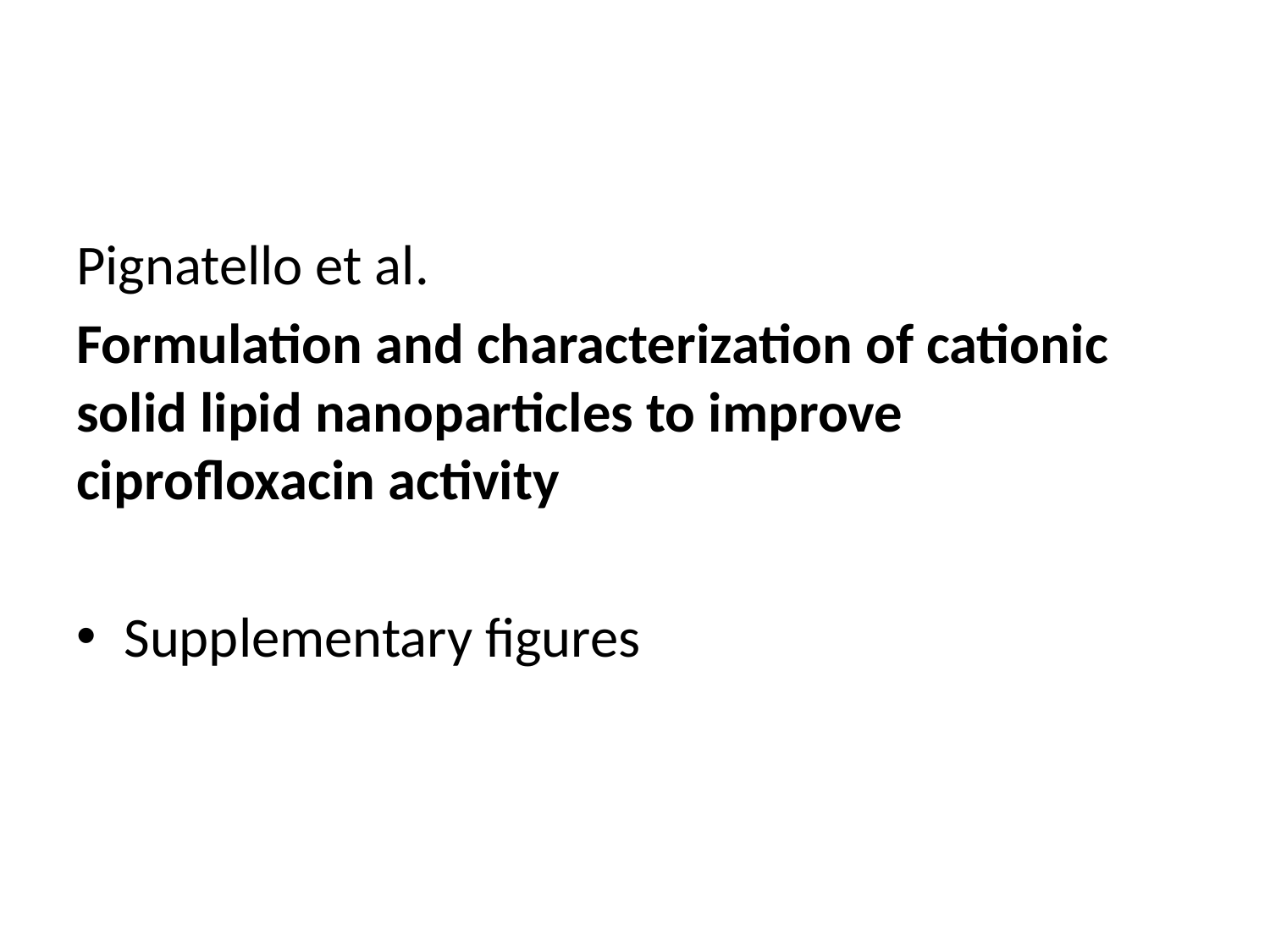

Pignatello et al.
Formulation and characterization of cationic solid lipid nanoparticles to improve ciprofloxacin activity
Supplementary figures

## Slide 2
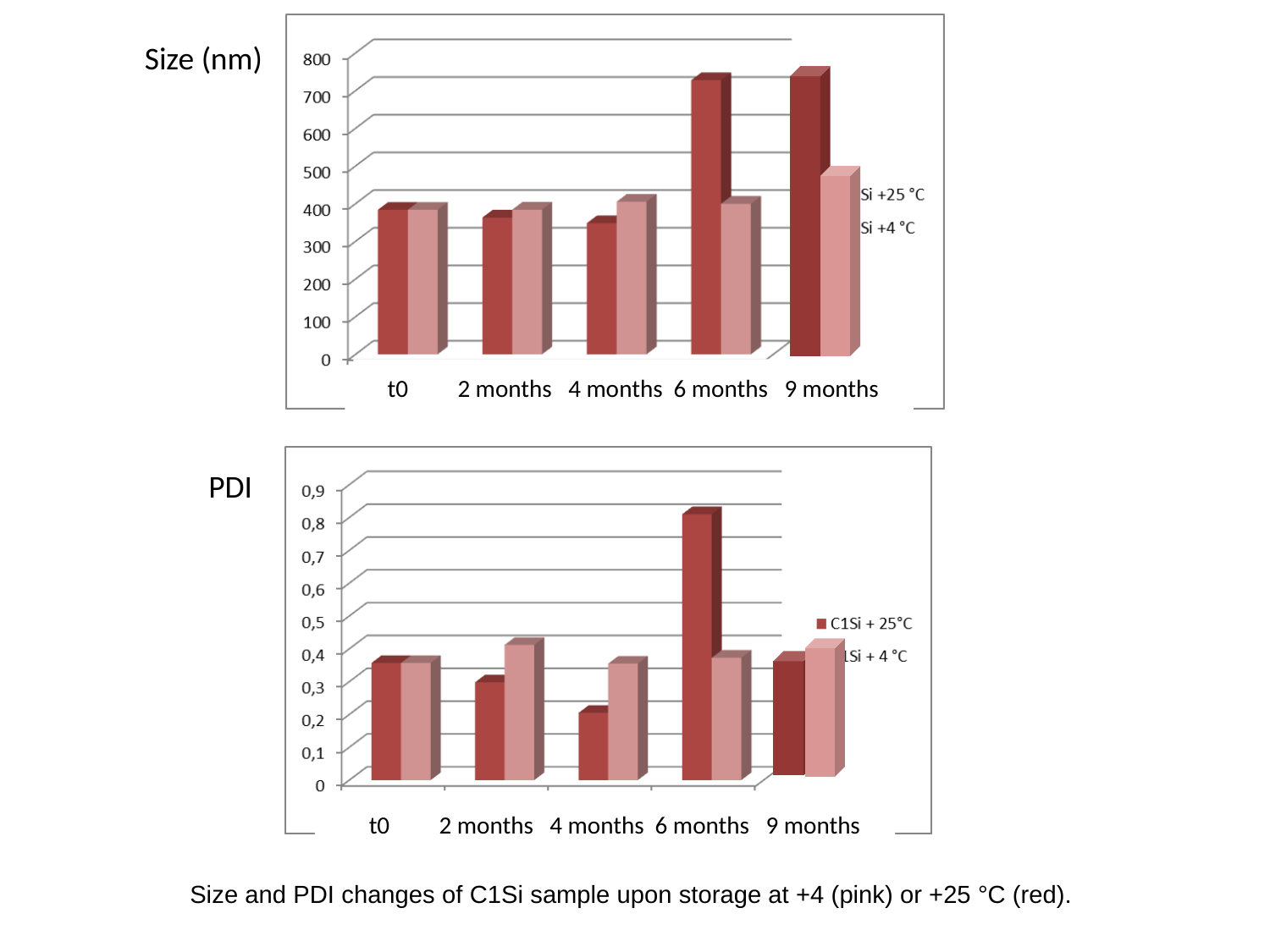

t0 2 months 4 months 6 months
Size (nm)
 t0 2 months 4 months 6 months 9 months
 t0 2 months 4 months 6 months
PDI
 t0 2 months 4 months 6 months 9 months
Size and PDI changes of C1Si sample upon storage at +4 (pink) or +25 °C (red).

## Slide 3
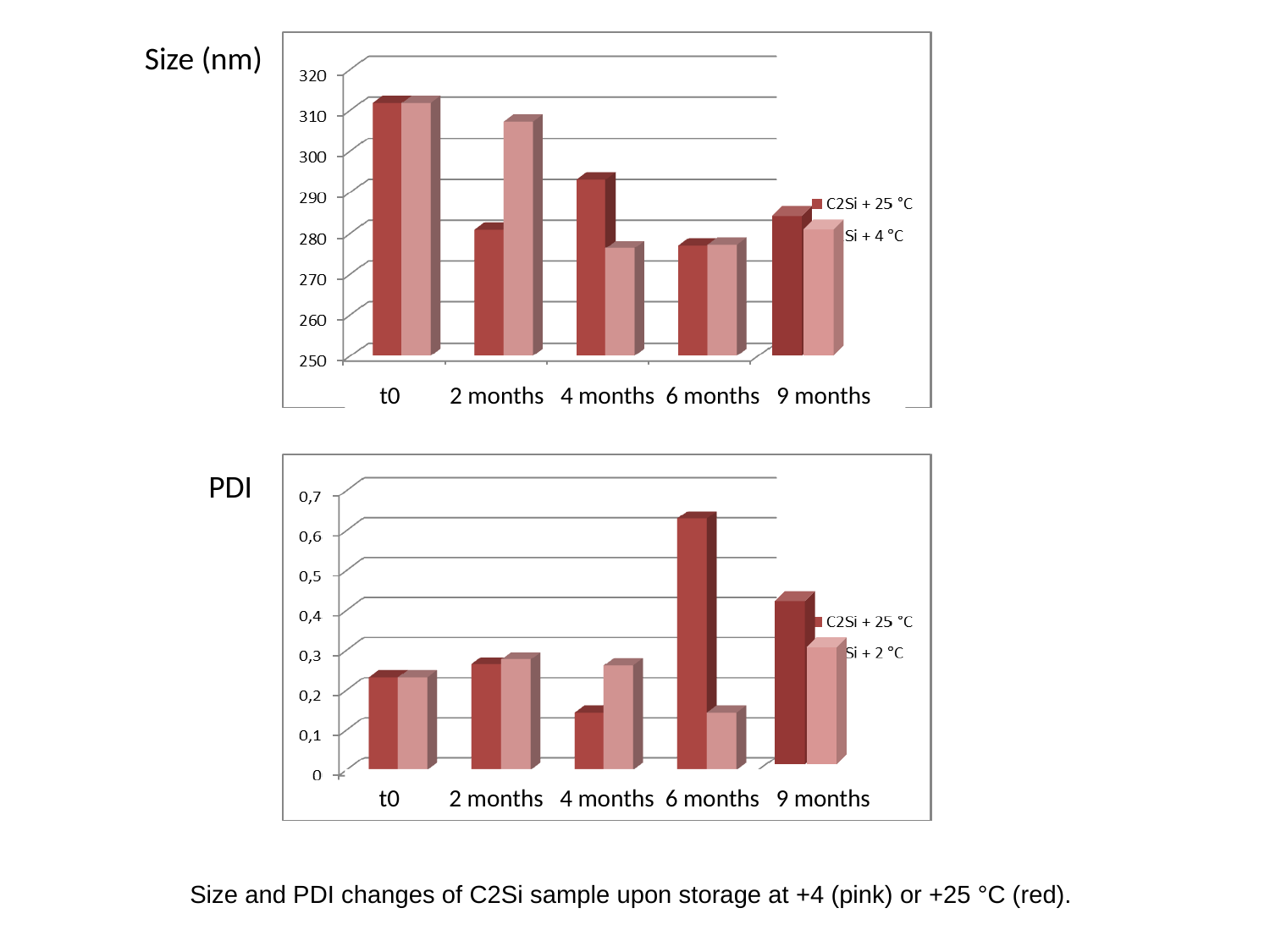

Size (nm)
 t0 2 months 4 months 6 months
 t0 2 months 4 months 6 months 9 months
 t0 2 months 4 months 6 months 9 months
PDI
Size and PDI changes of C2Si sample upon storage at +4 (pink) or +25 °C (red).

## Slide 4
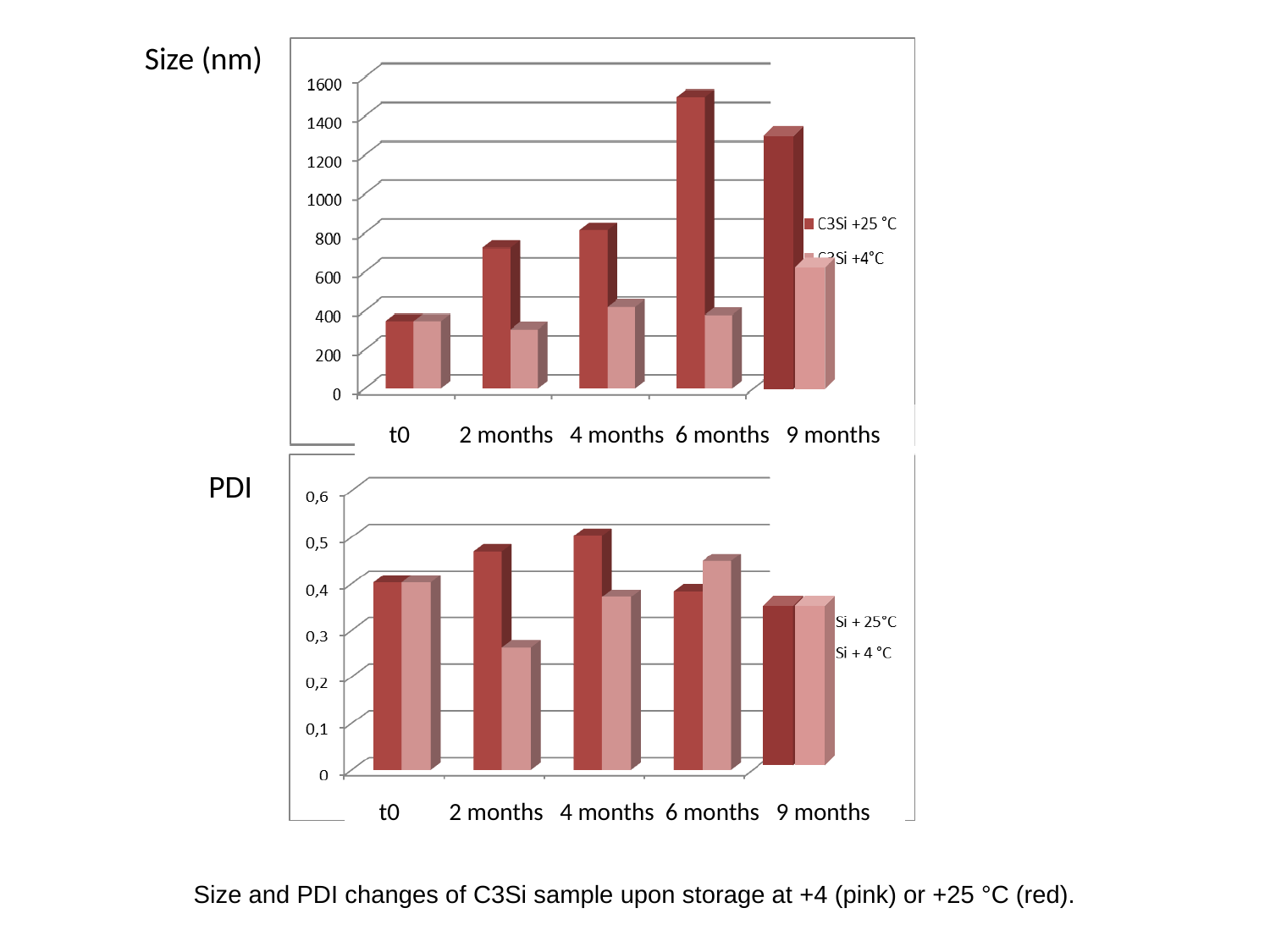

Size (nm)
 t0 2 months 4 months 6 months
 t0 2 months 4 months 6 months
 t0 2 months 4 months 6 months 9 months
PDI
 t0 2 months 4 months 6 months 9 months
Size and PDI changes of C3Si sample upon storage at +4 (pink) or +25 °C (red).

## Slide 5
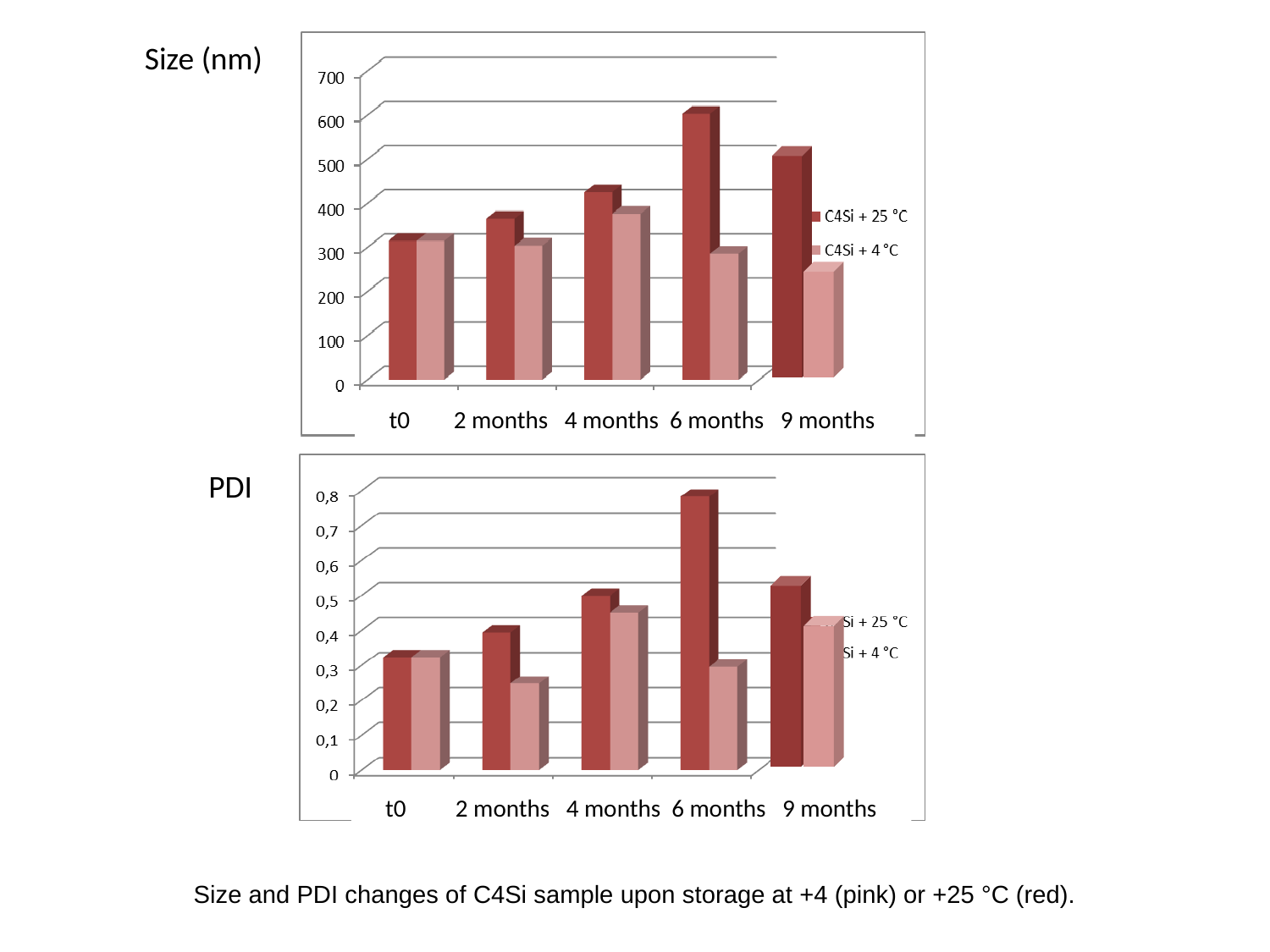

Size (nm)
 t0 2 months 4 months 6 months
 t0 2 months 4 months 6 months
 t0 2 months 4 months 6 months 9 months
PDI
 t0 2 months 4 months 6 months 9 months
Size and PDI changes of C4Si sample upon storage at +4 (pink) or +25 °C (red).

## Slide 6
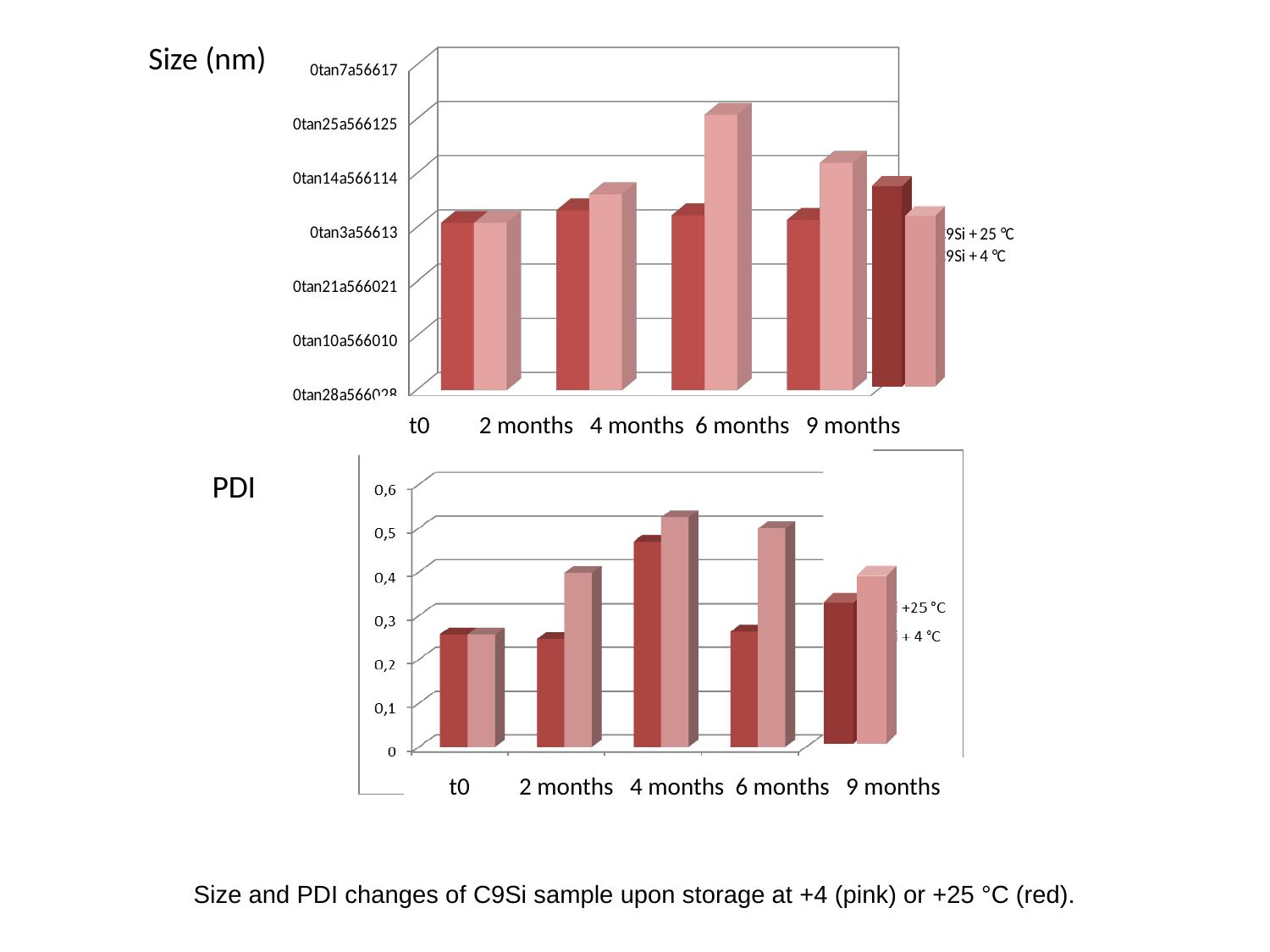

Size (nm)
[unsupported chart]
 t0 2 months 4 months 6 months
PDI
 t0 2 months 4 months 6 months
 t0 2 months 4 months 6 months 9 months
 t0 2 months 4 months 6 months 9 months
Size and PDI changes of C9Si sample upon storage at +4 (pink) or +25 °C (red).

## Slide 7
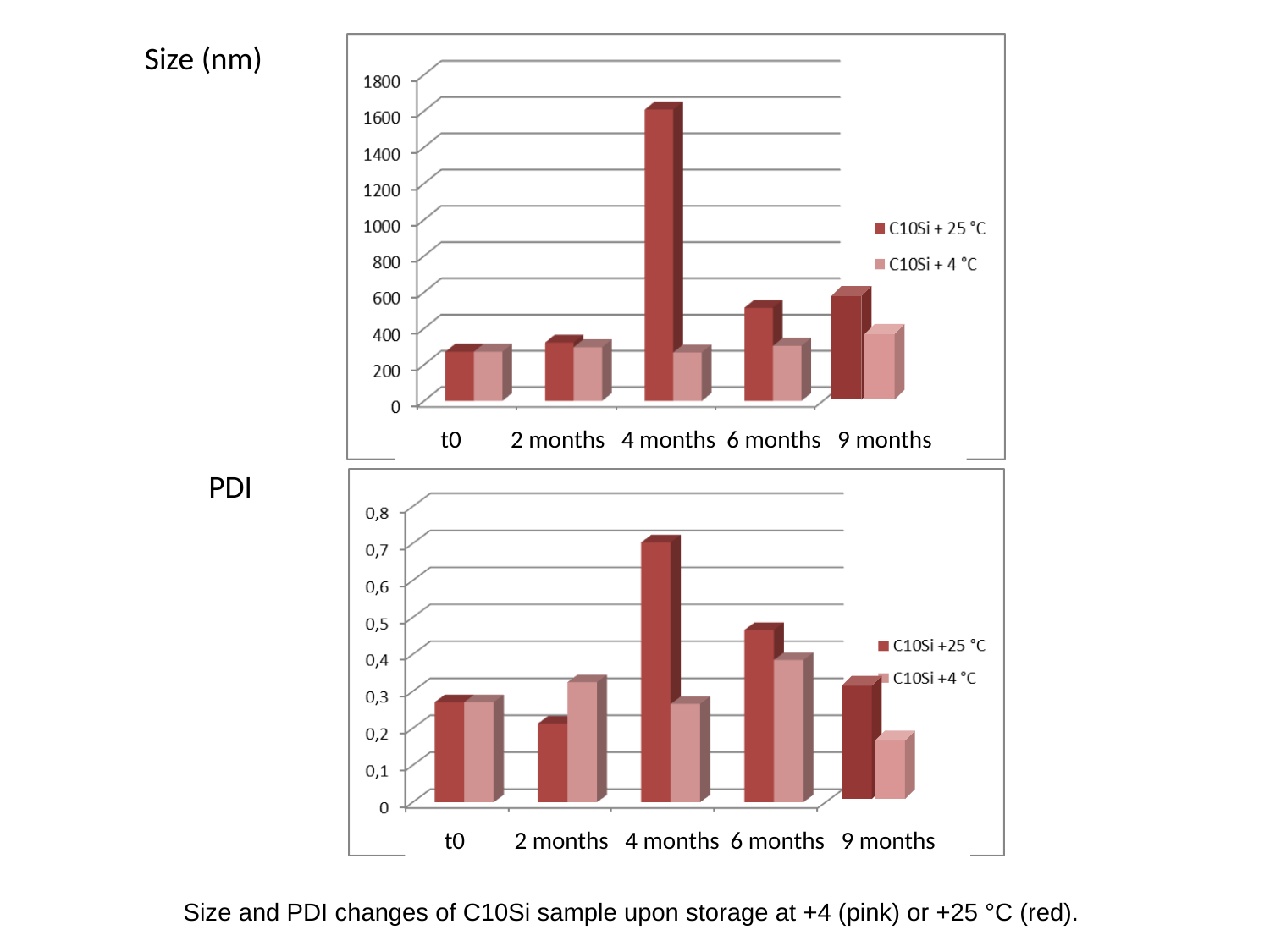

Size (nm)
 t0 2 months 4 months 6 months
PDI
 t0 2 months 4 months 6 months
 t0 2 months 4 months 6 months 9 months
 t0 2 months 4 months 6 months 9 months
Size and PDI changes of C10Si sample upon storage at +4 (pink) or +25 °C (red).

## Slide 8
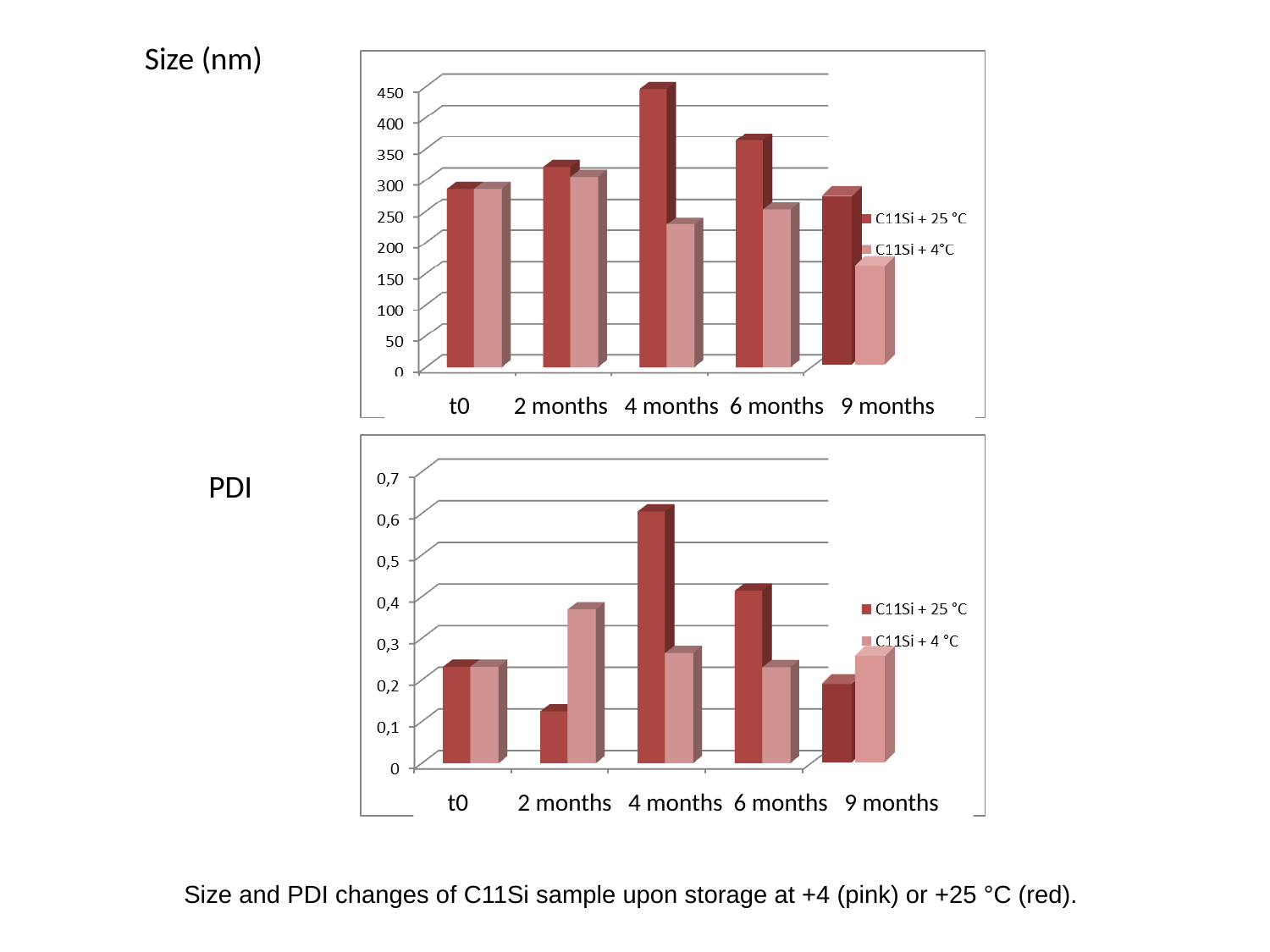

Size (nm)
 t0 2 months 4 months 6 months
PDI
 t0 2 months 4 months 6 months 9 months
 t0 2 months 4 months 6 months 9 months
 t0 2 months 4 months 6 months
Size and PDI changes of C11Si sample upon storage at +4 (pink) or +25 °C (red).

## Slide 9
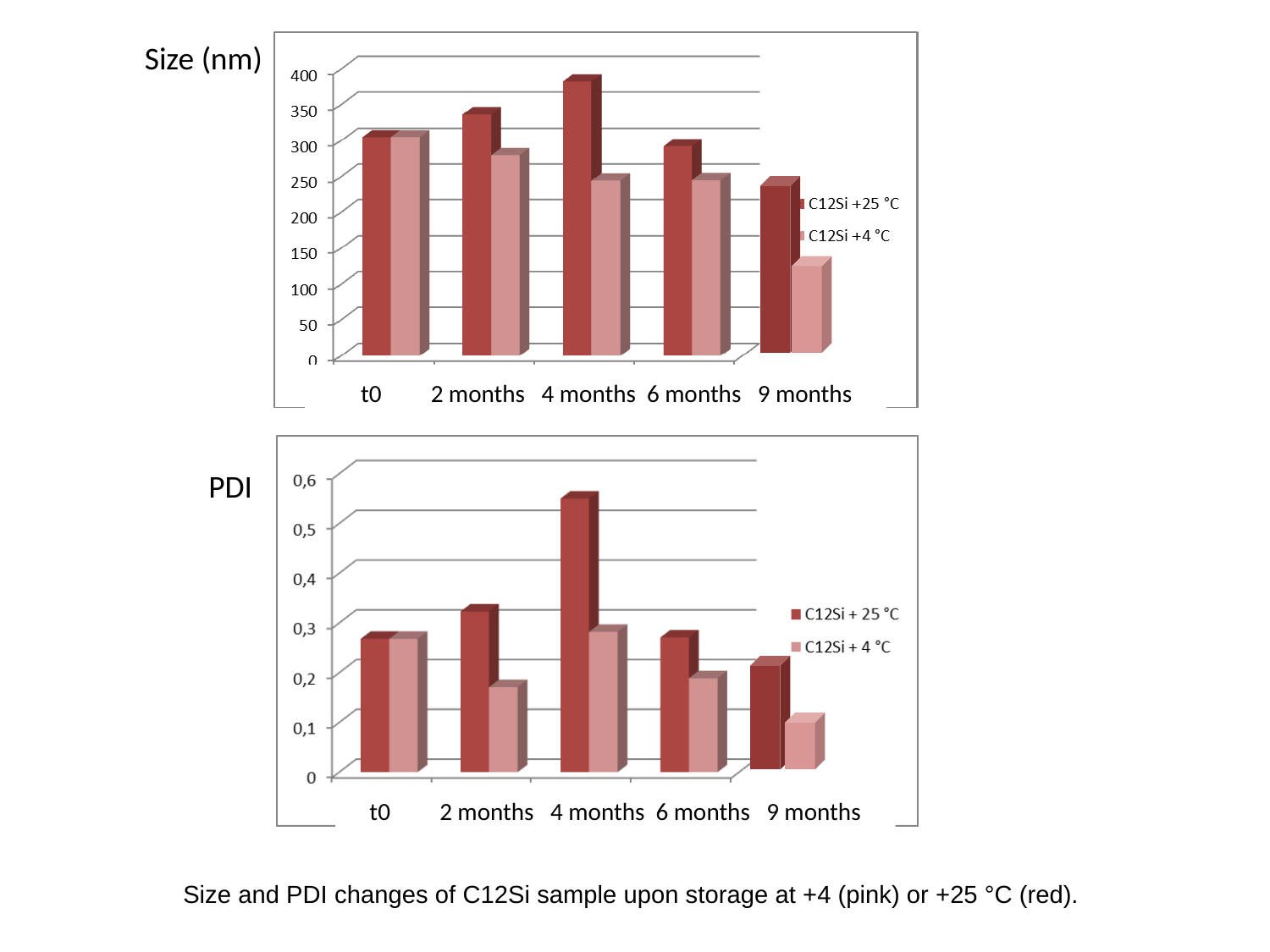

Size (nm)
 t0 2 months 4 months 6 months
PDI
 t0 2 months 4 months 6 months
 t0 2 months 4 months 6 months 9 months
 t0 2 months 4 months 6 months 9 months
Size and PDI changes of C12Si sample upon storage at +4 (pink) or +25 °C (red).
